# Combined electrocardiography, coronary angiography and magnetic resonance imaging for the diagnosis of viral myocarditis: A case report

**DOI:** 10.3892/etm.2014.1671

**Published:** 2014-04-07

**Authors:** JING ZHANG, SHENGHU HE, XIANG QI, YIMIN LI

**Affiliations:** 1 Department of Cardiology, Northern Jiangsu People’s Hospital, Medical College of Yangzhou University, Yangzhou, Jiangsu 225001, P.R. China; 2 Department of Cardiology, Nanjing Chest Hospital, Nanjing, Jiangsu 210000, P.R. China

**Keywords:** myocarditis, magnetic resonance imaging

## Abstract

Endomyocardial biopsy is the gold standard for diagnosing viral myocarditis. However, this method is rarely used as it is more invasive, less sensitive and has a higher incidence of complications than other methods. With recent developments in myocarditis research, cardiovascular nuclear magnetic resonance imaging has been demonstrated to have a marked advantage over endomyocardial biopsy, specifically regarding the differential diagnosis of acute coronary syndrome, as it is noninvasive, repeatable, highly sensitive and highly specific for diagnosing myocarditis. Myocardial edema is characteristic of myocardial inflammation, myocardial necrosis and myocardial fibrosis. T2-weighted nuclear magnetic resonance imaging sensitively detects myocardial tissue edema and additional imaging parameters contribute to the diagnosis of myocarditis. Therefore, combining these methods with the current sophisticated electrocardiogram and coronary angiography examination methods may facilitate the rapid and accurate assessment of viral myocarditis. A 44-year-old male patient with symptoms of dyspnea and shortness of breath accompanied by dizziness, through electrocardiography, coronary angiography and magnetic resonance imaging, was diagnosed viral myocarditis.

## Introduction

Myocarditis refers to a variety of myocardial inflammatory lesions. There are various causes of myocarditis, such as infection as well as physical and chemical factors, which result in myocardial damage of varying severity. The exact diagnosis of myocarditis requires histopathological evidence, which is predominantly obtained from endomyocardial biopsies. However, the diagnostic significance of endomyocardial biopsies during treatment is limited and is associated with certain surgical risks. Thus, it is not regularly used in current clinical treatments. The clinical manifestations of myocarditis differ due to variations in the degree of damage; thus, myocarditis is challenging to diagnose. For example, in a case where the history of viral infection is not clear and markers of myocardial necrosis are normal, the diagnosis of myocarditis is difficult to determine, regardless of clinical signs of heart damage, such as heart failure and arrhythmia. Therefore, a highly sensitive and accurate method is required to obtain a definitive diagnosis of myocarditis (1).

At present, cardiovascular magnetic resonance imaging (MRI) is an important tool in the noninvasive assessment of patients with suspected myocarditis, specifically in the differential diagnosis of acute coronary syndrome (ACS). Cardiovascular MRI is used for diagnosing myocarditis, in addition to guiding endomyocardial biopsies (2,3). The present study examines the importance of cardiovascular MRI in the diagnosis and differential diagnosis of myocarditis via a case report. This study was conducted in accordance with the Declaration of Helsinki and with approval from the Ethics Committee of Yangzhou University (Yangzhou, China). Written informed consent was obtained from the participant.

## Case report

**Figure 1:**
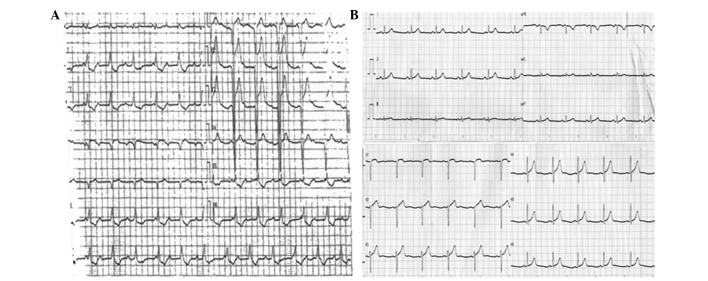
(A) Electrocardiogram (ECG) of the patient on admission, which was suggestive of acute anteroseptal and right ventricular myocardial infarction, ventricular escape and third degree atrioventricular block. (B) The patient was discharged following observation of a normal ECG.

A 44-year-old male patient was admitted to a local hospital one week subsequent to first presenting with symptoms of dyspnea and shortness of breath accompanied by dizziness, without severe chest pain, amaurosis or syncope. The electrocardiogram (ECG) demonstrated acute anteroseptal, right ventricular myocardial infarction, ventricular escape and a third degree atrioventricular block (AVB; Fig. 1A). Echocardiography showed mild mitral regurgitation and indicated that the cardiac structure, systolic function and wall motion were without abnormalities. The left ventricular ejection fraction was 65%, and the levels of troponin (Tn) were significantly increased. The patient was treated with an antiplatelet agent, an anticoagulant agent, vasodilators and a plaque stabilizer, and was transferred to the Northern Jiangsu People’s Hospital (Yangzhou, China) upon stabilization of the condition. The patient had previously been healthy, with a history of smoking (smoking index, 20 packs/year), but no history of hypertension, diabetes, hyperlipidemia, alcoholism or coronary heart disease. Furthermore, the patient had no recent history of upper respiratory tract infection or gastroenteritis virus.

The results of the physical examination of the patient were: Body temperature, 36.5°C; heart rate, 62 bpm; respiratory rate, 18 breaths/min; and blood pressure, 120/70 mmHg. The patient was conscious, exhibited no lip cyanosis and had a soft neck with no jugular vein engorgement. Clear breath sounds were heard from both lungs, with no rales and the patient exhibited a negative hepatojugular reflux. In addition, no abnormal precordial bulge was observed and the apex beat was at the fifth intercostal space, 0.5 cm medial to the left midclavicular line. The heart rate was regular. No pathological murmurs or pericardial friction rubs were identified on auscultation and the patient was negative for any peripheral vascular signs. The abdomen was soft, with no tenderness or rebound tenderness, and no pitting edema was observed on either of the lower extremities.

**Figure 2:**
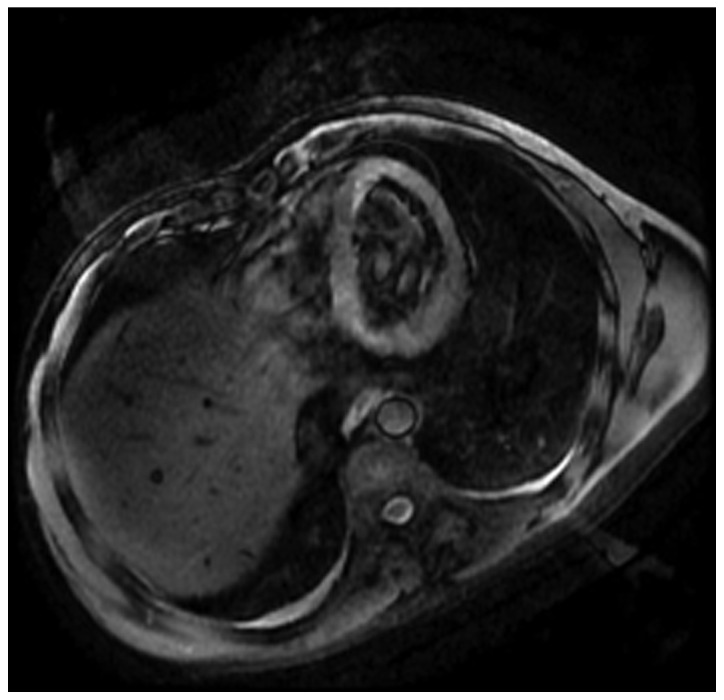
Patient observed using cardiovascular magnetic resonance imaging, the circle indicates the left ventricular anterior wall where a long T2 signal was observed.

The Tn level was 0.053 ng/ml (normal range, 0–0.034 ng/ml). Urinalysis, thyroid function, liver and kidney function were normal, as were the levels of blood glucose, blood lipids and electrolytes. Four days following admission to hospital, coronary angiography was conducted. This revealed normal openings and a running area without calcification in a right-dominant type coronary system in which: the left main coronary artery was without stenosis and had a forward flow of thrombolysis in myocardial infarction (TIMI) level 3; the left anterior descending had no significant stenosis and a forward flow of TIMI level 3; the left circumflex had no significant stenosis and a forward flow of TIMI level 3; the first obtuse marginal openings had visible plaques and a distal blood flow of TIMI level 3; and the right coronary artery had no significant stenosis, a forward flow of TIMI level 3 and no collateral circulation. On the basis of the imaging results, the diagnosis was coronary atherosclerosis. Six days following admission to hospital, the cardiac MRI revealed small, long T2 signals on the left anterior ventricular wall, which indicated slight edema (Fig. 2). The patient was prescribed bed rest to improve myocardial metabolism, traditional Chinese medicine to alleviate heart qi deficiency, and symptomatic treatment. The repeat ECG at 1 week after patient discharge was identified to be normal (Fig. 1B) and the symptoms of the patient had improved. Therefore, the patient was discharged.

## Discussion

The cardiovascular MRI of the patient indicated small, long T2 signals on the left ventricular anterior wall. This, in combination with the clinical manifestations and auxiliary examinations, such as the ECG, resulted in a diagnosis of myocarditis. Myocardial edema is characteristic of myocardial inflammation, myocardial necrosis and myocardial fibrosis, and can be detected via cardiac MRI (4). T2-weighted imaging is able to sensitively detect myocardial tissue edema (5), which emits a high intensity T2 signal. Furthermore, T2-weighted imaging characterizes myocardial damage in the acute phase of eosinophilic myocarditis as a signal of notably high intensity (6). Patients with myocarditis may present with an extensive myocardial edema; thus, it may be necessary to quantitatively analyze the signal intensity to improve the accuracy of the diagnosis (7–9).

In 1999, the China Society of Cardiovascular Disease developed the adult acute viral diagnostic reference criteria as follows: i) Influenza, diarrhea and other viral infections occurred within three weeks of a cardiac event. ii) Arrhythmias or ECG changes observed within three weeks of infection, including the following; a) ventricular tachycardia, AVB, sinoatrial or bundle branch block, b) multi-source, paired ventricular premature atrial or ventricular flutter or fibrillation, and c) two or more horizontal or downward sloping lead ST segments ≥0.01 mV, ST segment elevation or abnormal wall motion. iii) Myocardial injury reference or wall motion abnormalities; a) significantly increased troponin T (TnT), troponin I (TnI) and creatine kinase-MB levels, b) ultrasound cardiography showing heart chamber enlargement, and c) radionuclide examination confirming left ventricular systolic heart function or diastolic dysfunction. iv) The etiological criteria for diagnosing myocarditis are as follows: 1) A virus, a virus gene fragment or a viral antigen in the endocardium, myocardium, pericardium or a pericardial puncture. 2) Antibody isotypes to the same pathogenic virus detected over two weeks in the serum, with the first determination four-fold higher than the second, or an antibody titer ≥640. 3) Virus-specific immunoglobulin M levels ≥1:320 are considered positive. Patients are clinically diagnosed with myocarditis if they exhibit any one of the criteria in 1 and 2 or any two of the criteria in 1, 2 and 3, excluding other symptoms. Etiological diagnosis is based on the clinical diagnosis, which includes one of the items among the criteria in iv. Severe viral myocarditis may be observed in patients with Adams-Stokes syndrome, heart failure or ECG changes, including myocardial infarction, cardiogenic shock, acute renal failure, ventricular tachycardia with hypotension or cardiac pericarditis as one or more of the manifestations.

The majority of clinicians consider that conducting a endomyocardial biopsy is the gold standard for diagnosing myocarditis. The Dallas criteria describes lymphocyte infiltration with myocardial cell injury, but without myocardial ischemia; it has a high degree of specificity but a sensitivity of only 10–22% (American Heart Association, 1984). Endomyocardial biopsies result in a greater number of complications, such as cardiac perforation and cardiac tamponade occurring in ~0.1–0.5% of patients, in addition to a variety of complications with an overall incidence rate of ~6% (American Heart Association, 1984). The low sensitivity and risks that are associated with performing endomyocardial biopsies limit its use in the diagnosis of myocarditis; therefore, it is unsuitable for numerous patients with myocarditis, particularly those with mild myocarditis.

At present, cardiovascular MRI is an important tool for the noninvasive assessment of patients with suspected myocarditis (specifically in the differential diagnosis of ACS), and is useful for guiding endomyocardial biopsies. Lurz *et al* (10) identified that cardiovascular MRI has a sensitivity of 81%, specificity of 71% and accuracy of 81% for diagnosing acute myocarditis. The authors also demonstrated that the sensitivity, specificity and accuracy of cardiovascular MRI for diagnosing acute myocarditis were significantly higher than for diagnosing chronic myocarditis. Cardiac MRI is safe and reliable, and is capable of examining cardiac structure and function and performing myocardial perfusion scans in addition to other one-stop, accurate quantitative evaluations. Cardiac MRI directly identifies the characteristic pathological changes in myocardial tissue with a high repeatability; thus, it has become an internationally accepted cardiovascular imaging modality. In addition, cardiac MRI is highly sensitive and moderately specific for diagnosing myocarditis. Focal myocardial edema on T2-weighted imaging highlights the limitations of high signal enhancement scanning, as it appeared to strengthen with early myocardial involvement in delayed enhancement scanning. The shape and position of the delayed enhancement aids with distinguishing between the primary disease and ischemic cardiomyopathy, with the former strengthening in the myocardium or epicardium located in the subendocardial region. Focal myocardial enhancement and segmental wall motion abnormalities that occur simultaneously are indicative of myocarditis.

Cardiac MRI parameters exhibit differential precision. The T2 rate (assessment of edema), general myocardium-associated enhancement and late gadolinium enhancement (LGE) are specific parameters for detecting irreversible myocardial damage. Šramko *et al* (11) demonstrated that early gadolinium enhancement had a sensitivity of 40%, specificity of 96% and accuracy of 76% for the diagnosis of myocardial inflammation, whereas LGE had a sensitivity of 87%, a specificity of 44% and accuracy of 60%. Delayed enhancement scanning is of significant value in the diagnosis of myocarditis. Safiullina *et al* (12) investigated 51 patients with inflammatory cardiomyopathy and observed myocardial areas of delayed enhancement in 20 patients. Di Bella *et al* (13) reported observations for 81 patients in whom acute myocarditis was diagnosed by delayed contrast-enhanced MRI.

Patients with myocardial injury usually demonstrate elevated markers, abnormal Q waves or ST segment changes in their ECG. However, myocardial infarction and myocarditis are complex to diagnose in atypical cases with no medical history or symptoms. Furthermore, when diagnosing myocardial infarction in younger patients, other diagnostic possibilities should be excluded. In such patients, coronary angiography or coronary computed tomography angiography are recommended to exclude coronary artery disease. When the coronary arteries of a patient are healthy, cardiac MRI may be considered, with signs of edema in T2-weighted imaging or evident focal high signal enhancement enabling a definitive diagnosis of myocarditis. Combined electrocardiography, coronary angiography and magnetic resonance imaging is a favorable method for the diagnosis of viral myocarditis, and it requires further cases to confirm.
